# Paediatric intestinal pseudo-obstruction: a scoping review

**DOI:** 10.1007/s00431-021-04365-9

**Published:** 2022-04-28

**Authors:** Susan Nham, Alexander T. M. Nguyen, Andrew J. A. Holland

**Affiliations:** 1 Liverpool Hospital, Liverpool, NSW Australia; 2 South West Sydney Clinical School, The University of New South Wales, New South Wales, Australia; 3 The Burns Unit, The Children’s Hospital at Westmead Burns Research Institute, Westmead, NSW Australia; 4 Douglas Cohen Department of Paediatric Surgery, The Children’s Hospital at Westmead Clinical School, The Faculty of Medicine and Health, The University of Sydney, Corner Hawkesbury Road and Hainsworth Street, Westmead, NSW 2145 Australia

**Keywords:** Chronic pseudo-obstruction, Intestinal dysmotility, Paediatric, Children

## Abstract

Paediatric intestinal pseudo-obstruction (PIPO) encompasses a group of rare disorders in which patients present with the clinical features of bowel obstruction in the absence of mechanical occlusion. The management of PIPO presents a challenge as evidence remains limited on available medical and surgical therapy. Parenteral nutrition is often the mainstay of therapy. Long-term therapy may culminate in life-threatening complications including intestinal failure-related liver disease, central line thrombosis and sepsis. Intestinal transplantation remains the only definitive cure in PIPO but is a complex and resource-limited solution associated with its own morbidity and mortality. We conducted a scoping review to present a contemporary summary of the epidemiology, aetiology, pathophysiology, diagnosis, management and complications of PIPO.

*Conclusion:* PIPO represents a rare disorder that is difficult to diagnose and challenging to treat, with significant morbitity and mortality. The only known cure is intestinal transplantation.

**Table:** 

**What is Known:** *• Paediatric intestinal pseudo-obstruction is a rare, heterogeneous disorder that confers a high rate of morbidity and mortality* *• Complications of paediatric intestinal pseudo-obstruction include chronic pain, small intestine bacterial overgrowth and malrotation. Other complications can occur related to its management, such as line infections with parenteral nutrition or cardiac side effects of prokinetic medications*	
**What is New:** *• Progress in medical and surgical therapy in recent years has led to improved patient outcomes* *• Enteral autonomy has been reported in most patients at as early as 1 month post-transplantation*

**What is Known:**

*• Paediatric intestinal pseudo-obstruction is a rare, heterogeneous disorder that confers a high rate of morbidity and mortality*

*• Complications of paediatric intestinal pseudo-obstruction include chronic pain, small intestine bacterial overgrowth and malrotation. Other complications can occur related to its management, such as line infections with parenteral nutrition or cardiac side effects of prokinetic medications*

**What is New:**

*• Progress in medical and surgical therapy in recent years has led to improved patient outcomes*

*• Enteral autonomy has been reported in most patients at as early as 1 month post-transplantation*

## Introduction

### Background

Paediatric intestinal pseudo-obstruction (PIPO) encompasses a group of diseases involving the gastrointestinal neuromusculature [1]. The European Society for Paediatric Gastroenterology, Hepatology and Nutrition (ESPGHAN) defines PIPO as the “chronic (persisting for 2 months after birth or otherwise for 6 months or longer) inability of the gastrointestinal tract to propel its contents mimicking mechanical obstruction, in the absence of any lesion occluding the gut [2]”. The term “intestinal pseudo-obstruction” was first described in 1958 by Dudley et al. in adults who presented with these clinical findings and later in eleven children in 1977 by Byrne et al. [3, 4] Until recently, the diagnostic criteria for PIPO were poorly defined and a paucity of data still exists with regards to its epidemiology. Now understood to be a separate entity from chronic intestinal pseudo-obstruction in adults, PIPO is a congenital disorder in up to 80% of cases whilst acquired forms are rare. With the advent of genomic sequencing, some familial forms have been identified; however, most cases are sporadic [5]. Consequently, PIPO has been under-recognised leading to delays in diagnosis and challenges in management, with patients suffering both morbidity and mortality despite advances in technology and medical practices.

### Review objectives

The authors conducted a scoping review of the literature over the past 20 years on the topic of PIPO and present a summary of the aetiology, pathophysiology, diagnosis, management and complications of PIPO.

## Methods

### Search strategy

The Preferred Reporting Items for Systematic Reviews and Meta-Analyses (PRISMA) guidelines were followed [6]. An electronic search of the Cochrane, EMBASE and PubMed databases was conducted with review of articles from 2000 to September 30, 2020. Key words utilised included “children”, “paediatric”, “pediatric”, “pseudo-intestinal obstruction”, “chronic pseudo-intestinal obstruction” with appropriate Boolean operators (see Appendix). Reference lists of selected papers were also hand searched for further relevant articles.

### Inclusion criteria and selection of studies

Articles for analysis and inclusion in this paper were selected based on the following inclusion criteria: (1) meta-analyses, systematic reviews, randomised control trials, literature reviews, prospective and retrospective cohort studies, cross-sectional studies, case series and case reports; (2) article content focusing on the epidemiology, aetiology, pathophysiology, diagnosis, management and/or complications of intestinal pseudo-obstruction in children (both CIPO and PIPO); (3) age of patients between 0 and 18 years, (4) article written in the English language. Full-text versions of 248 articles after initial screening were read by two authors (SN, AN) independently; 189 articles were excluded as they did not fulfil the inclusion criteria. Disagreements were resolved by consensus.

## Results

**Fig. 1:**
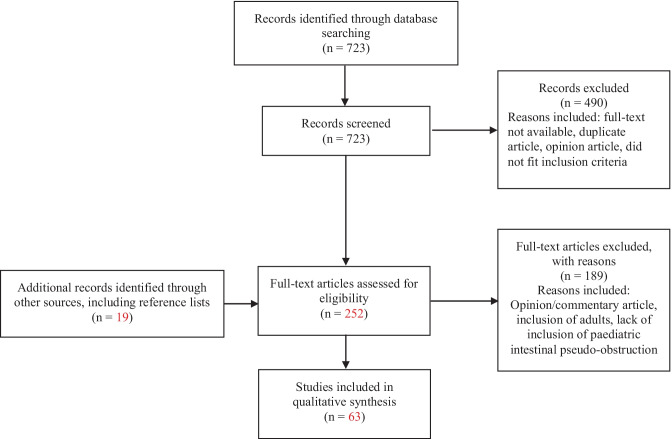
Literature search flow chart

In our initial literature search, we identified 723 articles. We screened the titles and abstracts of these papers and elected to remove 490 articles as they were either opinion articles, did not have an available full text or were duplicates. Subsequently, we reviewed the 248 full-text articles (which included 15 articles from reference list search) and included 61 articles in this scoping review. The high number of articles from reference lists reflected the articles we may have missed on screening an initially very large pool of search results. A flow diagram with results of search strategy is provided in Fig. 1. Table 1 summarises the study designs of the included studies.

**Table 1:** Study characteristics of the articles (by reference number) included in this systematic review

**Study type**	**Evidence hierarchy**[7]
5 Case reports: [8–12]	N/A
10 Case series: [13–22] 2 Cross-sectional: [23, 24]	Level IV
3 Case control: [25–27] 12 Retrospective cohort studies: [28–39] 1 Prospective cohort study: [40]	Level III-2
25 Review articles: [1, 2, 5, 41–63]	N/A
1 Systematic reviews, meta-analyses: [64, 65]	Level 1

## Discussion

### Epidemiology

The incidence and prevalence of PIPO is largely unknown. In 2014, Muto et al. conducted the first nationwide survey of PIPO in Japan, which found the prevalence to be 3.7 in 1 million in children less than 15 years of age. More than half of these children (56.5%) had developed PIPO in the neonatal period [24]. Other studies of nationwide surveys, including data from the American Pseudo-obstruction and Hirschsprung’s Disease Society, estimate that approximately 100 infants are born with PIPO in the USA each year [41, 66].

Overall, PIPO is a rare disease with a paucity of epidemiological data. According to the ESPGHAN, the incidence of PIPO is possibly less than 1 in 100 000. It is unclear whether an association exists between PIPO and geographical factors, ethnicity or sex. Similarly, data on mortality is hard to encounter — ESPGHAN’s estimates suggest that the mortality varies between 4.8 and 32%, and almost all deaths resulted from iatrogenic complications [2].

### Aetiology and pathophysiology

PIPO can be acquired or congenital with the aetiology classified into primary, secondary or idiopathic causes [42]. Up to 80% of cases are congenital whereas secondary forms of PIPO are rare and present in less than 10% of patients [2]. On histopathology, the aetiology of primary PIPO can also be divided into neuropathies, myopathies or mesenchymopathies [42]. Some may have normal histopathological findings, as demonstrated in 90% of children in Muto et al.’s survey. In contrast, only 10% had normal histopathological findings as reported by Waseem et al. [1, 24] Furthermore, as the findings in PIPO are varied, it is also not surprising that more than one type of pathology can be found on histology [43]. Whilst familial forms of PIPO can be inherited in an autosomal dominant, recessive or X-linked fashion, the majority of cases are sporadic [5]. The various causes of PIPO and known genetic associations are summarised in Table 2.

**Table 2:** Causes of PIPO identified [2, 8–11, 14, 42, 45, 46, 48–50]

Primary	Secondary
- Sporadic forms of neuropathy, myopathy and/or mesenchymopathy - Familial forms of PIPO -Autosomal dominant • SOX10 • ACTG2** - Autosomal recessive • RAD21 • SGOL1 • TYMP • POLG • LMOD1 - X-linked • FLNA • L1CAM • Fabry disease - Other • TTC7A deficiency	- Metabolic • Mitchondrial cytopathies - Autoimmune • Systemic lupus erythematosus • Scleroderma • Dermatomyositis • Polymyositis • Autoimmune myositis/leiomyositis • Autoimmune ganglionitis • Eosinophilic ganglionitis • Coeliac disease • Crohn’s disease - Infectious/post-infectious • Chagas’ disease • Cytomegalovirus • Herpes zoster virus • Epstein-Barr virus • Kawasaki’s disease • Post-viral neuropathy • Human immunodeficiency virus - Endocrine • Diabetes mellitus • Hypoparathyroidism • Hypothyroidism • Phaeochromocytoma • Multiple endocrine neoplasia IIb - Oncology/haematology • Chemotherapy and/or bone marrow/stem cell transplantation • Ganglioneuroblastoma (paraneoplastic) • Small cell carcinoma (paraneoplastic) • Sickle cell disease • Multiple myeloma - Toxins • Foetal alcohol syndrome • Jellyfish envenomation - Muscle disorders • Myotonic dystrophy • Duchenne muscular dystrophy - Neuropathies • Intestinal neuronal dysplasia • Autonomic neuropathies - Drugs • Diltiazem, nifedipine • Cyclopentolate/phenylephrine eye drops • Narcotics • Muscle relaxants - Developmental • Delayed maturation of ICC - Miscellaneous • Ehlers-Danlos syndrome • Amyloidosis • Radiation injury

*ACTG2*, actin gamma 2, smooth muscle; *FLNA,* filamin; *L1CAM*, L1 cell adhesion molecule; *LMOD1*, Leiomodin 2; *POLG*, polymerase DNA gamma; *RAD21*, cohesion complex component; *SGOL1*, shugoshin-like 1; *SOX10*, SRY-BOX 10; *TYMP*, thymidine phosphorylase

^**^There is evidence to suggest an autosomal recessive mode of inheritance also exists in some forms of PIPO with identified ACTG2 mutations

#### Enteric neuropathy

Enteric neuropathies of PIPO can be further subclassified into degenerative or inflammatory forms [44]. The enteric nervous system (ENS) within the gastrointestinal tract consists of the myenteric (Auerbach’s) and submucosal (Meissner’s) plexuses, which are responsible for producing regular muscular contractions via the migrating motor complex (MMC), preventing small intestinal bacterial overgrowth (SIBO) by propagating and clearing secretions, waste and microorganisms [43, 45]. PIPO resulting from disorders of the ENS are encompassed either by hypoganglionosis (or progressive loss) of enteric neurons or inflammatory processes [43].

In neurodegenerative PIPO, intestinal hypoganglionosis can be detected as early as during gestation and is characterised by low acetylcholinesterase activity, a reduction in the number of ganglion cells per millimetre length of bowel, two or fewer ganglion cells per ganglion, and distances between ganglion cells twice as far apart compared to healthy bowel [1, 45]. Qualitative histopathological findings also include degeneration of axons, neuronal swelling and other lesions [46]. Examples of neurodegenerative conditions causing PIPO include neuronal intranuclear inclusion disease, which also affects both the central and peripheral nervous systems and leads to other clinical features including ataxia, dysautonomia and dementia and diffuse intestinal ganglioneuromatosis, which is associated with multiple endocrine neoplasia type IIb and type 1 neurofibromatosis and results in the development of hamartomas of benign ganglion cells [45].

Inflammatory neuropathies, such as inflammatory enteric ganglionitis, affect the ENS ganglia, enteric neurons and neuronal connections [45]. Often associated with autoimmune conditions, infections and paraneoplastic syndromes, it is manifested by an infiltration of the ENS plexuses by predominantly CD3 + lymphocytes and, occasionally, plasma cells [44]. Even more uncommonly, the release of inflammatory mediators by enteric neurons can result in eosinophilic and mast cell ganglionitis. Clinical improvement is observed with immunosuppression; however, little is known about this clinical entity due to its rare incidence [47]. Left untreated, inflammatory enteric ganglionitis leads to neuronal degeneration and the eventual complete loss of ganglia [44].

#### Enteric myopathy

Enteric myopathies are often the result of genetic and/or congenital abnormalities, involve other viscera and confer a poorer prognosis compared to neuropathic PIPO [45]. Abnormalities in muscular layering associated with PIPO can be diffuse or focal [43]. In the muscular propria, which consists of the perpendicularly-aligned externa (longitudinal) and interna (circular) layers, the synchronised contraction and relaxation of each layer promote effective peristalsis of GIT contents. In focal or segmental disease, the muscularis propria retains its structural integrity; however, an additional muscle coat is found between the muscularis interna and the muscularis mucosa. This pathological finding is more commonly associated with the colon and results in severe segmental dilatation. In more diffuse disease, which tends to involve the small intestine, bundles of smooth muscle are abnormally layered in the muscular propria and dispersed between sections of normal bowel [45].

Mitochondrial myopathies, secondary to mutations in mitochondrial or nuclear genes affecting oxidative phosphorylation, are also becoming increasingly recognised. Of note, mitochondrial neurogastrointestinal encephalopathy (MNGIE) is a well-described autosomal recessive condition resulting from mutations in the thymidine phosphorylase gene, leading to impaired intestinal motility and also involving other organs [45, 47].

#### Mesenchymopathy

Disorders within the interstitial cells of Cajal (ICC) network have been reported in PIPO. ICC are considered the pacemaker cells of the GIT and are present in the submucosal and intramuscular layers [45]. Abnormal ICC, either in quantity or quality, have been associated with PIPO [43]. On immunohistochemistry, the presence of ICC is detected by their expression of c-kit receptors, which is required for the development of ICC. Significantly reduced c-kit positive cells in the myenteric plexus and muscularis propria have been associated with impaired GIT contractility and transit [13]. Of interest, ICC deficiency in neonates may represent a delayed process of ICC maturation: some presenting with signs and symptoms of PIPO are initially found to have an absence of c-kit on immunohistochemistry but later develop normal distributions in ICC with subsequent improvements in motility [47].

### Neonatal PIPO clinical features and diagnosis

Antenatal detection can occur in 20% of cases on sonography. This is manifested predominantly by megacystis; however, other findings include hydronephrosis, polyhydramnios and, more rarely, dilated bowel. Following birth, half to two-thirds of patients present within the first month of age, and most (approximately 80%) will have presented by 12 months [2, 28, 43, 46, 51, 62]. The remaining cases of PIPO sporadically develop in subsequent years of childhood [2].

In neonatal-onset PIPO, the clinical picture of acute episodes is predominated by abdominal distension, vomiting, constipation and delayed passage of meconium [2, 24, 29]. Pain is infrequently observed but may be present in 30% of cases in later-onset (infantile) forms [24, 29]. A consequence of intestinal stasis is SIBO, further compounding abdominal bloating and resulting in diarrhoea and steatorrhoea, which alternates with episodes of constipation and is seen in 30% of patients. In between acute episodes, patients can be asymptomatic or continue to experience these abdominal symptoms [50]. Dehydration and malnutrition are chronic complications secondary to malabsorption, anorexia and early satiety as oral intake tends to aggravate abdominal symptoms. These complications, which affect the growth and development of patients during a critical period, are under-recognised as true weights may be masked by third-spacing of fluid within distended bowel [2, 52].

### Late onset PIPO clinical features and diagnosis

The presentation of PIPO is wide-ranging in symptom type and severity. Whilst some patients will report only minor symptoms, others can have very severe and potentially intractable episodes. Malrotation and urinary tract involvement are conditions commonly associated with PIPO. Urinary tract involvement is present in 33% to 92% of cases, are largely secondary to visceral myopathy and includes urinary retention, recurrent urinary tract infections, hydronephrosis and vesicoureteric reflux [2, 52, 53]. The clinical entity of megacystis microlon intestinal hypoperistalsis syndrome (MMIHS) is detected antenatally by an enlarged bladder in 88% of cases [43]. Malrotation may be present in 28 to 36% and the diagnosis of PIPO should be considered if symptoms persist despite surgical correction for this [51].

The diagnosis of PIPO is challenging and time-consuming due to the rare nature of the disease and the need to exclude other common causes such as mechanical obstruction. Türer et al. reports the delay in diagnosis can range from 2 months to 2 years, in part as a result of an unclear and variable diagnostic criteria [15]. More recently, as per ESPGHAN, the diagnosis of PIPO requires a minimum 2 out of 4 of the following criteria in the absence of a mechanical cause of obstruction [2]:

- Small intestinal neuromuscular involvement (measured by manometry, histopathology, transit studies)
- Recurrent and/or persistently dilated loops of small intestine with air fluid levels
- Genetic and/or metabolic abnormalities associated with PIPO
- Inability to maintain adequate nutrition and/or growth on oral feeding (requiring supplemental enteral or parenteral nutrition)

The investigations below are recommended for the diagnosis of PIPO. Though not all are required for the diagnosis of PIPO, they remain recommended to understand the pathophysiological neuromuscular features, which can guide management and provide valuable prognostic information [52].

#### Laboratory investigations

Laboratory investigations are widely performed and can be investigated for secondary causes of PIPO. Generally, a full blood count, electrolytes, liver function tests, vitamin B12, cortisol levels, thyroid function tests, inflammatory markers and autoimmune screen are performed to identify reversible causes [2, 43, 52].

With most cases of PIPO being sporadic and very few associated genetic mutations identified thus far, genetic testing is likely to be of low diagnostic yield. The ESPGHAN recommends genetic work up to be reserved for extremely rare instances of PIPO, whereby other syndromic phenotypes or congenital abnormalities are also present [2]. In such cases, testing for specific abnormalities, as outlined in Table 2, would enable a patient-tailored approach to management and genetic counselling for families with risk-stratification and screening for recurrence in future pregnancies [2, 45]. Gamboa and Sood also suggest genetic testing for those presenting during the neonatal period whereby a sporadic genetic mutation may be identified and provide prognostic benefit to patients [43]. Beyond diagnostic purposes; however, genetic testing for research purposes may allow for genetic mutations and their phenotypes to be elicited, with future implications for avoidance of invasive assessments such as manometry.

#### Radiographs

The initial workup for these patients would include abdominal imaging to rule out a mechanical bowel obstruction. An erect or lateral decubitus abdominal radiograph may reveal air fluid levels and small bowel dilatation [43]. Axial imaging, such as computed tomography (CT) with contrast or magnetic resonance imaging (MRI) are preferred imaging modalities to identify intra- and extra-luminal causes of mechanical obstruction [2, 49, 53]. Small bowel follow-through studies are readily available, however, no longer commonly used due to the availability of CT, higher radiation dose and inability of patients to consume large quantities of water-soluble contrast [53].

#### Transit studies

[13^C^]-labelled acetate and octanoic acid breath tests provide an accurate measure of gastric emptying [49]. Well-tolerated and non-invasive, an abnormal result would highly suggest an underlying gastric motility disorder. Other intestinal transit studies may also have some diagnostic values but interpretation of these studies may be suboptimal due to the presence of SIBO [2].

#### Manometry

Manometry provides diagnostic information about the GIT neuromusculature and prognosticates response to treatment. As PIPO almost always involves the small bowel, antroduodenal manometry (ADM) gives highest diagnostic yield; however, the neuromuscular function of the oesophagus, colon, rectum and anus can also be investigated for extent of involvement, which guides management (such as multi-visceral transplantation) in severe cases [2, 43]. Enteric neuropathy is characterised by disorganised contractions with normal amplitude, which is distinguishable from myopathic forms, characterised by coordinated contractions with reduced amplitude [43, 52]. The presence of phase III MMC on manometry is a positive predictor of response to prokinetics and tolerance to feeds, whereas its absence or reduction in amplitude confers higher mortality and reliance on parenteral nutrition [51]. A normal manometry can exclude PIPO and should prompt consideration of factitious disorders [52].

#### Histopathology

Surgery tends to be avoided where possible due to the increased susceptibility for children with PIPO to form adhesions and have prolonged post-operative recovery [2]. Furthermore, a gastric lavage or prolonged fasting period is required pre-operatively to reduce aspiration risk because of delayed gastric emptying. If undergoing a surgical procedure, full-thickness biopsies can be obtained concurrently. Otherwise, the indication for histopathological assessment remains controversial — there is no pathognomonic finding for PIPO, histopathological findings rarely change management, and some patients show normal histopathology [2, 53]. As discussed in “Aetiology and pathophysiology”, the specific findings in PIPO include hypoganglionosis, neuropathies, ICC abnormalities, degenerative or structural disorders of the muscularis propria and mitochondrial abnormalities [42, 49, 53]. Zenzeri et al. and Di Nardo et al. recommend that full-thickness biopsies are indicated in (1) idiopathic cases of acute onset, or in (2) patients with rapidly progressive PIPO who have not responded to therapy and are not taking opiates, in which case a laparoscopic surgery is preferred [52, 53]. Endoscopic procedures are useful to exclude mechanical obstruction, and gastric and duodenal biopsies can be taken to rule out differentials [2].

### Management

The goals of treatment are to preserve bowel function, optimise nutrition and growth, improve quality of life, whilst minimising complications of supportive treatment and the need for unnecessary surgical intervention. Many patients will require parenteral feeding; however, the aim would be to wean this where possible to achieve enteric autonomy. A multidisciplinary team is required involving paediatric gastroenterologist, surgeon, dietitian, psychologist, geneticist, social worker and other allied health personnel [43]. Though rare, if a treatable cause of PIPO is identified, management is aimed at treating the underlying disease [42].

#### Nutritional support

Various strategies to maintain nutrition in patients with PIPO include oral feeding, enteral nutrition (EN) and parenteral nutrition (PN) [2]. Up to two-thirds of patients will require PN during the disease course; however, in the long-term, one-third of patients will tolerate oral nutrition, another third will require EN and the remaining third will require partial or total PN [51, 54]. Poorer outcomes for intestinal autonomy are associated with neonatal-onset PIPO and the presence of urinary tract involvement [55].

In patients who are able to tolerate oral nutrition, small frequent meals with liquids, soft foods and low-fibre multivitamin supplementation are encouraged and can improve intestinal motility [49, 52, 53]. Foods high in carbohydrates and fats should be avoided as they can worsen abdominal bloating [52].

EN is initiated in children who are unable to meet their nutritional needs with oral feeds alone [5]. EN is preferable in those with the presence of MMC activity as it maintains stimulation of the GIT mucosal transporters, preserves GIT architecture and function and promotes mucosal growth [5, 49, 54]. EN is initially administered via a nasogastric tube as constant or cyclical feeds [52]. In many cases, especially in those with evidence of delayed gastric emptying, the stomach is bypassed with naso-jejunal or percutaneous endoscopic jejunostomy (PEJ) tubes [49, 52, 53].

PN can be commenced alone or in combination with EN if enteral feeds fail or are inadequate. PN is considered total PN (TPN) when it provides 100% of the daily caloric needs [5]. In attempts to improve quality of life for patients, the delivery of patient-tailored home PN has been introduced, which have been shown to be well-tolerated and minimises hospitalisation, however, require extensive family education [54].

##### Complications of PN

Although lifesaving, PN is associated with a number of serious complications, including intestinal failure associated liver disease (IFALD) and central venous catheter (CVC)-related complications. A study by Mousa et al. determined that up to 90% of deaths were due to PN-related complications rather than the underlying disease itself [30].

CVC-related complications encompass thrombosis and sepsis secondary to line infections. Venous access for PN is obtained via one of six large veins and if more than half are lost, there is a significant risk of losing access completely, which in turn hinders the potential process of intestinal transplantation [56].

IFALD exists as a spectrum from initial cholestasis, with variable fibrosis and steatosis, to irreversible cirrhosis [25, 56]. Due to the lack of diagnostic criteria, the prevalence is unknown with Pironi et al. reporting its presence in 15 to 85% of those receiving home PN [55]. IFALD arises from a combination of SIBO bacterial translocation, recurrent line sepsis, release of endotoxins and synthetic concentrations of PN solutions with excessive glucose and lipids and inadequate micronutrients and amino acids [16, 57]. Steatosis develops initially due to hepatic accumulation of lipids and glycogen. This is followed by cholestasis, which results from the aforementioned factors, and is further compounded by biliary stasis and impaired gallbladder contractility in the absence of enteral feeding. This combination of steatosis and cholestasis and the accumulation of toxic bile acids are manifested initially by elevations in bilirubin and transaminases. Although asymptomatic in the early stages, jaundice may be observed in some patients. If left untreated, hepatic dysfunction can progress to irreversible cirrhosis. In advanced disease, complications of portal hypertension and synthetic function arise, resulting in coagulopathy and thrombocytopenia [57, 63]. In a cross-sectional study on the deleterious long-term effects of PN on IFALD, Mutanen et al. found that although biochemical markers of liver function may normalise after weaning of PN, abnormal histology may persist for up to 9 years [25]. As such, the greatest concern for mortality in patients receiving PN is the progression of IFALD to liver failure. Consequently, efforts should be made to wean off PN when commenced before irreversible liver failure ensues [56]. Measures to reduce the risk of IFALD whilst receiving PN can be taken by altering the composition of the PN emulsion. Emulsions containing high amounts of omega-6 fatty acids have been associated with liver dysfunction, whereas emulsions containing pure fish oil (omega-3 fatty acids) have been shown to be effective as rescue therapy in those with severe liver disease. Concerns have been raised with long-term use of fish oil as the sole source of lipids due to its inability to meet fatty acid requirement. Consequently, a major advance in the delivery of PN is the use of mixed-lipid emulsions consisting of fish oil, soybean oil, coconut oil and olive oil. Mixed-lipid emulsions provide a better balance between omega-3 and omega-6 components and contain antioxidants in the forms of α-tocopherol and γ-tocopherol, which reduce cholestasis and limit hepatic toxicity [55, 57].

#### Pharmacotherapy

Pharmacotherapy aims to manage SIBO, improve symptoms and promote gastrointestinal motility [52]. There is limited evidence supporting the use of pharmacotherapy in the management of PIPO to improve gastrointestinal motility with nil-recommended treatment for most patients. Medications in the form of prokinetics, antibiotics and probiotics are often trialled at the discretion of the clinician [2].

##### Prokinetics

Serotonergic agents, cisapride (combined 5-HT4 agonist and 5-HT3 antagonist), tegaserod (5-HT4 agonist) and prucalopride (selective 5-HT agonist) are the most well-studied prokinetics that have demonstrated improvements in gastrointestinal motility and improved enteral tolerance [2, 45, 53]. These medications bind to serotonergic receptors to promote post-ganglionic acetylcholine release in the myenteric plexus, thereby increasing smooth muscle contraction and, in turn, antroduodenal motility. Unfortunately, cisapride and tegaserod have been withdrawn due to increased risk of cardiac arrhythmias and adverse cardiovascular events [45, 49]. More promisingly, prucalopride has been generally well-tolerated in children and adults with minimal adverse effects due to its selective action on 5-HT4 receptors located predominantly in the large and, to a lesser extent, small bowels. A recent systematic review on prucalopride, which included case reports of PIPO with recurrent episodes and/or poor response to other medical management, demonstrated improvements in abdominal distension, nausea and vomiting and increased tolerance with oral feeds [65].

Octreotide, a somatostatin analogue, induces intestinal phase III of the MMC and accelerates transit time within the small bowel [53]. It has been shown to relieve SIBO in adults with scleroderma-induced chronic intestinal pseudo-obstruction and increase tolerance of enteral feeding in small studies in children with PIPO [31, 53]. It is postulated that octreotide may have a synergistic effect with erythromycin, which mimics motilin and evokes gastric phase III MMC, overcoming the inhibitory effects of octreotide on gastric emptying [49, 53]. The efficacy of erythromycin has not been extensively investigated with concerns regarding tachyphylaxis, macrolide resistance and cardiovascular safety (including torsades de pointes and sudden cardiac death) [49, 51, 67].

Acetylcholinesterase inhibitors, such as neostigmine and pyridostigmine, increase the availability of acetylcholine at nicotinic and muscarinic receptors, stimulating smooth muscle contraction and increasing gastrointestinal motility [12, 17]. There have been reports of efficacy and good tolerance in children receiving intravenous neostigmine, including in the setting of PIPO secondary to haematological malignancies [12]. Oral pyridostigmine has only been trialled in a number of small case series and case reports at 0.25–4 mg/kg/day in divided doses with reported reductions in abdominal pain, distension, and gastric drainage, and increased tolerance to enteral intake [8, 17].

Dopamine receptor antagonists, metoclopramide and domperidone have antiemetic and prokinetic properties. Studies have reported improvements in oesophageal peristalsis and gastric emptying. However, these have not been well-studied with concerns for pseudo-Parkinsonism and tardive dyskinesia with chronic use of metoclopramide, and cardiac dysrhythmias with domperidone [49, 67].

##### Pain

Management of abdominal pain poses a considerable challenge and requires a multidisciplinary team including chronic pain and mental health specialists. Pain arises from gaseous distension of bowel loops secondary to the underlying intestinal dysmotility. However, this can be further exacerbated by concurrent SIBO which increases fluid secretion and gas production. Abdominal symptoms may only be experienced during acute episodes but can persist in between episodes [50]. Although paracetamol and non-steroidal anti-inflammatory drugs are suitable adjuvants, analgesia during acute episodes may often rely on opioids, which further disrupt intestinal motility [43, 45]. Buprenorphine has been considered in some case reports to be a better-tolerated and more effective alternative to morphine if side effects are of significant issue and pain remains inadequately controlled [18]. Non-opioid medications such as tricyclic antidepressants, gabapentin and pregabalin have also been trialled as monotherapy and adjuncts to good effect and low doses have been proposed for long-term use to manage chronic pain [45]. In cases where pain is not adequately controlled with analgesics, ostomy formation may be required to provide relief from symptoms (see below).

##### Small intestine bacterial overgrowth

SIBO has a detrimental effect on malabsorption and gastrointestinal motility by worsening abdominal distension and mucosal inflammation [43, 49, 52]. Management of SIBO often involves weekly to fortnightly therapy of combined broad-spectrum antibiotics and antifungal compounds, which are rotated with antibiotic-free periods [49]. Commonly used antibiotics include amoxicillin-clavulanic acid, metronidazole, sulphamethoxazole and trimethoprim, rifaximin and aminoglycosides [43, 52]. Amoxicillin-clavulanic acid has been shown to have additional prokinetic benefits in children [26], whilst rifaximin is minimally absorbed and may be the preferred option in minimising antibiotic resistance [53]. Vigilance should be exercised when prescribing pharmacotherapy as these drugs do not come without side effects. Liver function should be monitored as commonly used antibiotics, such as amoxicillin-clavulanate, may cause liver injury and hepatitis which can worsen outcomes for a population already prone to IFALD [2].

There is minimal evidence for the use of probiotics in SIBO. However, recent studies have demonstrated improvements in symptoms with faecal transplantation [2]. Common probiotics that have been trialled in the prevention of SIBO include *Lactobacillus casei*, *Streptococcus faecalis/Clostridium butyricum* and *Bifidobacterium longum/Bifidobacterium infantis*. Traditional Kampo medicines (daikenchuto and rikkunshito) have also been trialled in combination with probiotics with promising effects on intestinal motility and constipation. The postulated mechanisms relate to stimulation of the cholinergic pathways and interactions with serotonergic receptors including 5HT-3 and 5HT-4 [23].

#### Surgical therapy

The role of surgery is controversial as it has been associated with high post-operative morbidity and mortality. At the same time, due to variable and non-specific clinical presentations, coupled with (previously) ill-defined diagnostic criteria and lack of early recognition of PIPO, many patients will have undergone multiple surgical procedures to exclude mechanical obstruction. At baseline, patients with PIPO are more susceptible to developing adhesions and suffer prolonged post-operative recovery. Surgery itself is a risk factor for worsening prognosis and clinical symptoms, increasing the risk of subsequent adhesive small bowel obstruction, and thus, should be avoided if at all possible [15, 32].

Differentiating true mechanical causes of obstruction from an episode of PIPO poses another challenge. Clinicians should be suspicious of a surgical cause for a patient’s abdominal pain, distension and constipation when symptoms fail to improve with conservative management for PIPO. In particular, patients with PIPO are 10 times more likely to develop volvulus than their healthy counterparts — this may be due to a concurrent intestinal malrotation or as a resultant complication from dilated bowel loops [30]. de Betue et al. describe two such instances of potential life-threatening volvulus, which when identified and treated operatively, led to a rapid recovery of their patients’ abdominal symptoms [19].

Surgical procedures with the aim of forming a gastrostomy or enterostomy may be necessary for venting of distended gastrointestinal segments, facilitation of enteral feeds, and in certain emergencies such as severe bowel distension, perforation or ischaemia [2, 52]. Decompression of bowel is associated with a number of favourable outcomes in approximately 50% of patients including: reduction in symptoms of abdominal distension and vomiting, preservation of residual intestinal motor function, reduced SIBO, fewer septic episodes related to bacterial translocations. Venting procedures may be more effective in alleviating abdominal symptoms compared to pharmacotherapy and reduces reliance on PN by increasing tolerance of enteral feeding. Overall, improved symptomatology results in improved quality of life [2, 53]. Complication rates are higher in PIPO and include non-functioning stomas, peristomal inflammation, stomal prolapse and intestinal necrosis [27, 33]. A retrospective case control study of 44 children requiring stomas identified that stomal prolapse occurred in 45% of patients with PIPO compared to 9% in controls. Further surgical intervention was required in 60% with intestinal necrosis requiring resection occurring in 20% [27]. Although no risk factors for prolapse were identified in the study, the authors put forward the possibility of underlying neuropathy and/or myopathy as contributors to higher complication rates [27, 34]. Carefully constructed ostomies and diligence in stoma care can reduce the risk of complications [27]. Neuropathic PIPO confers a better prognosis and, more promisingly, in those who have been weaned and remain off PN for an extended period of time without major issues, closures of decompressive ileostomies have been achieved with 66% of children continuing on with oral and/or enteral feeding alone [2]. This supports the hypothesis of the self-limiting nature of some cases of PIPO that improve with maturation of the affected neurons [34].

#### Transplantation

The only definitive cure for PIPO is intestinal transplantation [2]. Transplantation should be considered when other medical and surgical therapies have failed in patients with severe complications of PN (including liver failure and recurrent central line sepsis), loss of central venous access for PN (two or fewer suitable veins remaining) and poor quality of life whilst receiving PN [2, 52, 58]. Dysmotility disorders represent the second-most common group requiring intestinal transplantation, making up to 25% of patients accepted onto the waiting list. Of these, PIPO makes up the most common subgroup [58]. With improving survival and quality of life associated with PN and venting ostomies, PIPO can be better managed without proceeding to transplantation. Due to the limited availability of resources and associated high mortality rates, intestinal transplantation is a last resort [2].

The types of intestinal transplants can vary depending on the extent of disease and presence of complications, as outlined by ESPGHAN [2]. As such, the assessment and workup for transplantation is extensive, involving manometry, assessment of liver disease and its reversibility via sonography, radiological imaging and potential biopsy [56]:

- Isolated intestinal transplant is considered for those with normal foregut motility, normal liver function or mild IFALD.
- Combined liver and bowel transplant is considered in normal foregut motility with moderate to severe IFALD.
- Modified multi-visceral transplant involves transplantation of the small bowel with the stomach, duodenum, pancreas and (in some cases) large bowel. This is indicated in patients with foregut dysmotility without significant IFALD.
- Multi-visceral transplantation is indicated for patients with foregut dysmotility with moderate to severe IFALD and encompasses the organs involved in a modified multi-visceral transplant with the additional liver transplant.

Patients with PIPO, in particular the MMIHS population, should also undergo a urological workup as a renal transplant may be warranted in those with end-stage renal failure [56].

The development of improved surgical approaches and better immunosuppressive therapy have increased overall survival and reduced graft rejection rates [2, 52]. Complications vary from surgical-related, such as wound infections, graft ischaemia, visceral perforations, intestinal obstruction, delayed gastric emptying, biliary tract dilatation; to transplant-related such as graft rejection and bacterial, fungal and viral opportunistic infections [52, 58]. Of the latter, monitoring for cytomegalovirus and Epstein-Barr virus infections is imperative to minimise predisposition to associated lymphoproliferative disorders [58].

Furthermore, the road to transplantation is not without issues. As evidenced by the intestinal transplant programme in Australia, only one-third of those referred were deemed suitable for transplantation, with the remainder being too unwell or having unsuitable co-morbidities. The rate of successful intestinal transplantation is limited by donor availability and the presence of donor-specific antibodies. As a result, a number of patients also died whilst waiting for a transplant. Thus, a fine delineation must be made between listing appropriate patients for transplantation, the criteria for which may already be life-threatening, before they become too unwell to proceed with the procedure [35].

Unfortunately, transplantations in children with dysmotility disorders carry a high rate of mortality. The United Network for Organ Sharing reported a 1- and 5-year survival rate of 75% and 57%, respectively [36]; whilst the intestinal transplant registry reported a 10-year survival rate of 30% amongst 1351 children [57]. Infection, graft rejection, multiorgan failure and lymphoma have been identified as common causes of mortality [20, 37, 59]. These complications arise from the delicate balance between adequate and excessive immunosuppression [58].

There have been reports of enteral autonomy being achieved in most patients at as early as 1 month post-transplant, although these statistics vary across studies, depending on transplant centre and immunosuppressive regime [58]. Goulet et al. report successful complete weaning of PN in 80% of cases after 3 years and partial dependence on PN in 7% [59], D’Antiga and Goulet report partial PN dependence rate of 40% [57], whilst other small cohort studies have observed over 90%, if not all, surviving patients to be off PN and tolerating oral and/or enteral feeds well [20, 21, 56, 60].

### Outcomes

PIPO carries a mortality rate of approximately 20%, with a number of observational studies reporting rates between 10 and 32% [24, 30, 39, 40]. Predictors of poor outcome include myopathic forms of PIPO, urinary tract involvement, oesophageal dysmotility and concurrent intestinal malrotation [30, 52, 61]. In particular, MMIHS has been associated with a particularly unfavourable prognosis with survival rates as low as 19.7%, the main causes of mortality being sepsis, malnutrition or multiorgan failure [64]. As previously discussed, for PIPO as an entity, up to 90% of deaths are associated with complications from PN, including IFALD and central line sepsis [30]. More recently, a literature review by Pironi et al. revealed that the 1-, 5-, and 10-year survival in children with PIPO on home PN were 90%, 70% and 60%, respectively [55]. Compared to intestinal transplantation, superior survival in patients on PN and supportive management highlight the paramount importance of exhausting all available avenues of management to avoid transplantation.

PIPO presents significant morbidity to children, who have a poorer quality of life as evidenced by reports of fewer pain-free days, and increased anxiety and depression compared to healthy children. Additionally, there is greater burden placed on their families and carers, requiring increased time and effort to care for them compared to healthy controls [38, 43]. Due to the challenges of diagnosing PIPO, many patients would have undergone multiple surgical procedures prior to the diagnosis being made, further compounding their clinical presentation and symptoms of pain, ileus and malnutrition [15, 32]. Survival rates have not been shown to differ between neonatal-onset and late-onset PIPO; however, retrospective cohort studies have observed trends of higher rates of dependence on PN (and hence, IFALD) and surgical procedures in the neonatal-onset population, whereas malnutrition was more evident in the late-onset form, which although more mild in severity, is a reflection of more chronic, complex and comorbid disease [24, 29].

### Future outlook

PIPO is a disorder that confers significant morbidity and mortality. Many challenges have arisen in understanding its pathophysiology and management. Due to its rare prevalence, most published studies are in the form of case reports, small case series and cohort studies. Nevertheless, these studies have given rise to improvements in medical and surgical therapy in recent years and have improved quality of life and survival in patients. The challenging nature of PIPO stems from under-recognition of this clinical entity, worsened by the lack of diagnostic criteria until recently. These criteria remain broad and PIPO has been shown to encompass many heterogenous disorders. Future research is required to better classify PIPO into more precise subtypes beyond histopathological findings. There may be benefit in providing more accurate epidemiological data in disease incidence and prevalence, and identification of further genetic mutations, which may shed light on various phenotypes of the disorder, and thereby elicit a better understanding of the underlying cellular pathophysiology. This, in turn, will pave the way for more effective medical and surgical management of this debilitating disorder. Of interest, a number of novel management approaches have emerged, including allogenic haematopoietic stem cell transplantation to restore thymidine phosphorylase function in patients with MNGIE [43, 45], and insertion of gastrointestinal electrodes that act as pacemakers to improve nausea and vomiting [42, 49]. These approaches are still in its infancy and require further studies to explore their efficacy and long-term outcomes.

## Conclusion

PIPO is a rare, debilitating disorder whereby existing therapy is limited, has variable efficacy and the potential to give rise to life-threatening complications. Whilst recent advances in medicine have improved the overall survival for patients with PIPO, mortality remains high and long-term quality of life remains restricted. Surgical procedures are judiciously indicated but may provide relief from symptoms of obstruction where medical therapy has failed. Importantly, they are associated with risks of significant complications. Thus, there is ongoing need for further research to investigate management strategies that are feasible, effective and definitive in relieving symptoms and preventing complications prior to considering transplantation, a resource-limited solution with impaired survival.

## Appendix: Literature search strategies

**Table:**

Search engine	Search terms	Number of results	Results removed	Results on initial screening
PubMED	(“pediatric” OR “paediatric” OR “children”) AND “intestinal pseudo obstruction”	284	107	177
PubMED	(“paediatric” OR “pediatric” OR “children”) AND “chronic pseudoobstruction"	129	27	102
EMBASE	(“paediatrics” OR “children”) AND “intestine pseudo-obstruction”	311	197	104
Cochrane	Pediatrics AND Intestinal pseudo-obstruction	0	0	0

## Abbreviations

ADM: Antroduodenal manometry
CIPO: Chronic intestinal pseudo-obstruction
CT: Computed tomography
CVC: Central venous catheter
EN: Enteral nutrition
ENS: Enteric nervous system
ESPGHAN: European Society for Paediatric Gastroenterology, Hepatology and Nutrition
ICC: Interstitial cells of Cajal
IFALD: Intestinal failure associated liver disease
MMIHS: Megacystis microlon intestinal hypoperistalsis syndrome
MMC: Migrating motor complex
MNGIE: Mitochondrial neurogastrointestinal encephalopathy
MRI: Magnetic resonance imaging
PIPO: Paediatric intestinal pseudo-obstruction
PN: Parenteral nutrition
PEJ: Percutaneous endoscopic jejunostomy
PRISMA: Preferred reporting items for systematics reviews and meta-analyses
SIBO: Small intestine bacterial overgrowth
TPN: Total parenteral nutrition
